# Accelerated Microbial Reduction of Azo Dye by Using Biochar from Iron-Rich-Biomass Pyrolysis

**DOI:** 10.3390/ma12071079

**Published:** 2019-04-02

**Authors:** Wenbing Tan, Lei Wang, Hanxia Yu, Hui Zhang, Xiaohui Zhang, Yufu Jia, Tongtong Li, Qiuling Dang, Dongyu Cui, Beidou Xi

**Affiliations:** 1 State Key Laboratory of Environmental Criteria and Risk Assessment, Chinese Research Academy of Environmental Sciences, Beijing 100012, China; wenbingtan@126.com (W.T.); wangleicraes@163.com (L.W.); zhanghuiguom@163.com (H.Z.); dangling819@126.com (Q.D.); cuidy@craes.org.cn (D.C.); 2 State Environmental Protection Key Laboratory of Simulation and Control of Groundwater Pollution, Chinese Research Academy of Environmental Sciences, Beijing 100012, China; 3 School of Life Sciences, South China Normal University, Guangzhou 510631, China; yhanxia@126.com; 4 State Key Laboratory of Vegetation and Environmental Change, Institute of Botany, Chinese Academy of Sciences, Beijing 100093, China; yufu123jia@163.com; 5 College of Resource Environment and Tourism, Capital Normal University, Beijing 100048, China; 18811785922@163.com

**Keywords:** Orange G dye, biochar, iron-rich-stalk pyrolysis, microbial reduction, electron transfer

## Abstract

Biochar is widely used in the environmental-protection field. This study presents the first investigation of the mechanism of biochar prepared using iron (Fe)-rich biomass and its impact on the reductive removals of Orange G dye by *Shewanella oneidensis* MR-1. The results show that biochars significantly accelerated electron transfer from cells to Orange G and thus stimulated reductive removal rate to 72–97%. Both the conductive domains and the charging and discharging of surface functional groups in biochars played crucial roles in the microbial reduction of Orange G to aniline. A high Fe content of the precursor significantly enhanced the conductor performance of the produced biochar and thus enabled the biochar to have a higher reductive removal rate of Orange G (97%) compared to the biochar prepared using low-Fe precursor (75%), but did not promote the charging and discharging capacity of the produced biochar. This study can prompt the search for natural biomass with high Fe content to confer the produced biochar with wide-ranging applications in stimulating the microbial reduction of redox-active pollutants.

## 1. Introduction

Synthetic chemical dyes are widely and frequently used in various modern industries, including textiles, leather, plastics, paper, printing, pharmaceuticals, food, and cosmetics [1,2]. Approximately one million tons of dyes are produced annually in the world, and over 10% of these dyes are discharged directly into the environment [3,4]. Azo dyes are the most important dyes and constitute about 70% of the whole dye family [5]. They are a class of molecules characterized by the presence of one or more azo bonds (–N=N–), which are often connected with aromatic rings and auxochromes (e.g., –SO_3_^−^ and –OH). Azo dyes are toxic, mutagenic, carcinogenic, and colored, and thus pose a significant risk to humans and ecological environments [6].

The removal of azo dyes from wastewater has been extensively studied to mitigate their threat to the environment. A wide range of physicochemical and biological treatment technologies have been developed and applied for the removal of various azo dyes from wastewater [7]. The physicochemical technologies mainly include adsorption [8,9,10], ozonation [11], Fenton treatment [12,13], and photocatalysis [14,15]. In biological technology, the azo bonds of azo dyes are split into small molecules with aromatic structures by using anaerobic microbial reduction and subsequently mineralized to CO_2_ under aerobic conditions [16,17]. No single technology is considered an absolute advantage in removing the azo dyes from wastewater. Thus, the development of new efficient technologies and materials for the treatment of azo dyes in wastewater is greatly needed and of practical interest.

Biochar is a carbon (C)-rich solid material formed by the incomplete combustion of diverse biomass species under an oxygen (O_2_)-absence condition. Until recently, studies on the impact of biochar on the fate of contaminants have focused on adsorption [18], where biochar is considered to be physically porous and chemically inert toward adsorbates [19]. However, biochar is not merely a passive adsorbent but a catalytically functional material that has the potential to mediate the abiotic reduction of toxic organic compounds [20]. Although the mechanisms regarding the reductive removal mediated by biochar are not fully understood, the catalytic ability of biochar appears to stem from its conductor and battery performance [21]. The conductor mechanism involves electron transfer through the conductive domains of biochar, whereas the battery mechanism involves the electron flux through the charging and discharging of the surface redox-active functional groups in biochar [22].

Analogous to the role of biochar in abiotic systems, biochar-mediated electron transfer has also been applied in biological systems [23], wherein biochar possesses the potential to stimulate microbial reduction of pollutants, such as nitrate and pentachlorophenol [20,21]. However, limited knowledge is available about whether biochar can stimulate the microbial reduction of azo dyes and whether both the battery and conductor performances of biochar can contribute to the microbial reduction of azo dyes. In addition, iron (Fe), which is ubiquitous in plant biomass, is a transition metal element with high electrical conductivity (EC). Fe-based chemical materials are increasingly produced and prevalently applied in environmental remediation [24]. Although Fe-doping treatment can lead to a high content of Fe in biochar, Fe is often not evenly distributed in biochar and high processing costs would be required. Thus, pyrolysis of Fe-rich biomass may be an alternative method for preparing biochar. However, whether the biochar from Fe-rich-biomass pyrolysis can enhance its conductor and battery performances and thus accelerate the microbial reduction of azo dyes has not been studied. Clearly, this question warrants investigation given its expected importance to promoting the functionality of biochars in a low-cost way.

This study used two different biochars, which were prepared using Fe-rich and Fe-poor peanut stalks as extracellular electron shuttles for the microbial reduction of Orange G (C_16_H_10_N_2_Na_2_O_7_S_2_) dye by *Shewanella oneidensis* MR-1 in the simulated wastewater. The main objectives of this study were (1) to assess the effects of biochars as battery and conductor on the microbial reduction of Orange G and (2) to test whether the biochar from Fe-rich-stalk pyrolysis can accelerate the microbial reduction of Orange G. The results from the present study can promote the further application of biochar from iron-rich-stalk pyrolysis on the microbial removal of redox-active pollutants and provide a theoretical guidance for promoting the industrialization of biochar.

## 2. Materials and Methods

### 2.1. Peanut Stalks Used for the Production of Biochars

Two types of peanut stalks (*Arachis hypogaea* L.), which were obtained from a plot without Fe addition and a plot with Fe addition (45 kg FeSO_4_ ha^−1^ y^−1^), respectively, in the same agricultural land, were selected for the production of biochars. As shown in Table 1, the Fe concentrations in the peanut stalks from the plot with Fe addition (PSWF) were significantly higher than those in the peanut stalks from the plot without Fe addition (PSNF). Notably, no remarkable differences were found in the contents of the elements C, nitrogen (N), hydrogen (H), and O between PSWF and PSNF.

### 2.2. Production and Chemical Analyses of Biochars

Both the dried PSNF and PSWF were cut into 4 cm chips. These stalk chips were pretreated with 1 M NaOH solution for 1 min to remove the biological residues on the surfaces of the stalks and then rinsed with deionized water to neutral and dried at 105 °C. The pretreated stalk chips of PSNF and PSWF were pyrolyzed in a muffle furnace (KJ-T1200-S10440-LK, Kejia Electric Furnace Co., Ltd., Zhengzhou, China) to produce biochars. The pyrolysis temperature was programmed to increase at a rate of 10 °C min^−1^ and be maintained at 600 °C for 2 h. The amount of stalks pyrolyzed and the yield of corresponding biochar in one batch were about 300 g and 100 g, respectively. The biochars produced using PSNF and PSWF were called BCNF and BCWF, respectively.

The total C, H, and N concentrations of the biochars were determined using a high-temperature automated elemental analyzer (vario EL cube, Elementar Company, Hanau, Germany). The O contents of the biochars were also determined using an elemental analyzer by using a catalyst of nickel (Ni, 30%)-impregnated C in the absence of O in the combustion atmosphere. The concentrations of Fe, phosphorus (P), potassium (K), calcium (Ca), magnesium (Mg), cobalt (Co), copper (Cu), zinc (Zn), molybdenum (Mo), and Ni in the biochars were determined using an inductively coupled plasma mass spectrometry (X Series, Thermo Fisher, MA, USA). The pH, ECs, and ash contents of the biochars were measured following the methods of a previous study [25]. The specific surface area of the biochars were determined using the method of Dai et al. [26].

### 2.3. Microbial Reduction Capacities of Biochars

*S. oneidensis* MR-1 was cultured according to the procedure of a previous study [27]. The culture after incubation for 24 h was centrifuged. The cell pellet was then washed three times with 30 mM NaHCO_3_ buffer (pH = 6.8) and suspended at a density of 10^10^ cells mL^−1^. Anoxic biochar suspensions were prepared following the method of Kappler et al. [28]. The final concentration of the biochar suspensions was 150 g L^−1^. The suspensions were dispersed using an ultrasonic probe for 10 min, degassed under vacuum for 2 min, flushed with N_2_ for 5 min, and sterilized using autoclave (120 °C for 20 min).

Microbial reduction assays were conducted in serum bottles containing 10 mL of 30 mM NaHCO_3_ buffer (pH = 6.8), sodium lactate (30 mM), biochar (7.5 g L^−1^), and *S. oneidensis* MR-1 cells (10^8^ cells mL^−1^). All incubation samples were prepared thrice in parallel. A total of 200 µL of culture suspension, after a microbial reduction of 96 h, was filtered (0.22 µm) and then reacted with 2 mL of 5 mM ferric iron complexed with citrate acid (Fe(III)-citrate) for 24 h, to quantify the microbial reduction capacities of the biochars. A total of 200 µL of the reaction mixture was added to 5 mL of 50 mM HEPES-buffered ferrozine (1 g L^−1^) solution. Absorbance was measured immediately using an ultraviolet-visible spectrophotometer (UV-1800, Shimadzu, Kyoto, Japan) at 510 nm.

### 2.4. Treatment Design and Incubation Experiments for the Removal of Orange G

The removal experiments of Orange G were set up in 100 mL serum bottles containing NaHCO_3_ buffer (30 mM, pH = 6.8) with a final volume of 80 mL. Eight treatments were performed with three replicates each: (I) Orange G was added to the prepared serum bottles; (II) Orange G and BCNF suspensions were added to the prepared serum bottles; (III) Orange G and BCWF suspensions were added to the prepared serum bottles; (IV) Orange G and *S. oneidensis* MR-1 suspensions were added to the prepared serum bottles; (V) Orange G, *S. oneidensis* MR-1 suspensions, and BCNF suspensions were added to the prepared serum bottles; (VI) Orange G, *S. oneidensis* MR-1 suspensions, and BCWF suspensions were added to the prepared serum bottles; (VII) Orange G and the BCNF suspensions, after a microbial reduction of 96 h by *S. oneidensis* MR-1 and subsequent sterilization through ultraviolet radiation, were added to the prepared serum bottles; (VIII) Orange G and the BCWF suspensions, after a microbial reduction of 96 h by *S. oneidensis* MR-1 and subsequent sterilization through ultraviolet radiation, were added to the prepared serum bottles. The initial concentration of Orange G in the treatments with additions of Orange G was 150 mg L^−1^. The initial concentration of the biochars (BCNF or BCWF) in the treatments with additions of biochars was 7.5 g L^−1^. The initial density of *S. oneidensis* MR-1 in the treatments with additions of cells was 10^8^ cells mL^−1^. Prior to the start of the incubation, all serum bottles were shaken to homogenize the suspensions, flushed with N_2_ to keep an anaerobic condition, and then sealed with butyl rubber stoppers and kept at room temperature (about 22 °C) in the dark throughout the 96 h incubation.

Treatment (I) was conducted as a control. Treatments (II) and (III) were conducted to assess the adsorbing removal of Orange G by biochars as the adsorbent. Treatment (IV) was conducted to assess the microbial reduction of Orange G by *S. oneidensis* MR-1. Treatments (V) and (VII) were conducted to assess the stimulated microbial reduction of Orange G by biochars as the conductor and multicycle battery. Treatments (VII) and (VIII) were conducted to assess the removal of Orange G by microbially reduced biochars as one cycle of the battery.

### 2.5. Sample Collection and Analysis

To assess the removal efficiencies of Orange G under different treatments, 3 mL of homogenized suspension sample in each serum bottle was withdrawn at different time points over 96 h. Each sample was divided into two subsamples, which were used for the determinations of Orange G and aniline, respectively. One of the subsamples was filtered using a 0.22 µm cellulose acetate membrane and then used for the determination of Orange G by using an ultraviolet-visible spectrophotometer (UV-1800, Shimadzu, Kyoto, Japan) at 478 nm. The other subsample was extracted using sonication for 15 min with 2 mL of dichloromethane. The solvent extracts were rotary evaporated to dryness, transferred into methanol, and filtered using a 0.22 µm cellulose acetate membrane. The concentration of aniline was measured using a high-performance liquid chromatography (Agilent 1260, Santa Clara, CA, USA) equipped with a C18 column (4.6 mm × 250 mm, 5 µm). A total of 30% ultrapure water in methanol was used as eluent (flow rate of 0.5 mL min^−1^). The injection volume was 20 µL, and the detector wavelength was 254 nm. The concentration of aniline was quantified using the external standard method, and the standard curve of the aniline was at a significant level (*R*^2^ > 0.999).

### 2.6. Calculations and Statistical Analysis

The removal rate of Orange G in each treatment was calculated based on the following equation:

(1) $$\mathrm{Removal}\;\mathrm{rate}\;\left(\%\right)=\left(1-\frac{C_{t}}{C_{0}}\right)\times 100$$

where *C*_0_ and *C_t_* are concentrations of Orange G in the solution at incubation times 0 and t, respectively.

Statistical analyses were conducted with SPSS software Version 18.0 for Windows. Data are represented as arithmetic mean ± arithmetic standard deviation, which were calculated from replicates. The significant difference was evaluated using one-way analysis of variance (ANOVA) at the level of *P* < 0.05. 

All chemicals used in this study were purchased from Sinopharm Chemical Reagent Co., Ltd (Shanghai, China).

## 3. Results and Discussion

### 3.1. Physicochemical Characterization of Biochars

**Figure 1:**
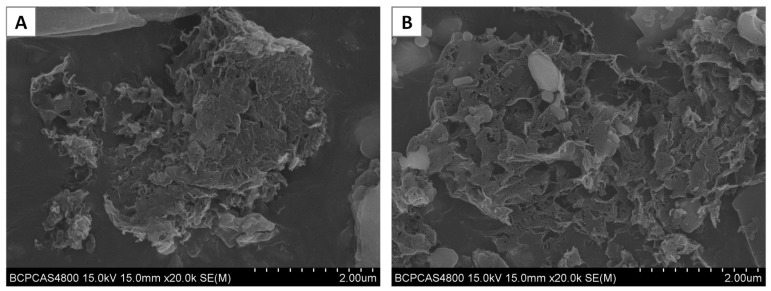
SEM images of biochars prepared using peanut stalks from the plot without Fe addition (**A**) and from the plot with Fe addition (**B**).

The SEM (Scanning Electron Microscope) images of BCNF and BCWF are shown in Figure 1, and their physicochemical properties are listed in Table 2. The Fe content was significantly higher in BCWF than those in BCNF, possibly due to the higher Fe content in PSWF than that in PSNF (Table 1). Except for the Fe content, other physicochemical properties shown in Table 2 did not show significant differences between BCNF and BCWF, possibly due to the fact that both biochars were prepared using peanut stalks.

### 3.2. Adsorbing Removal of Orange G with Biochar as the Adsorbent

**Figure 2:**
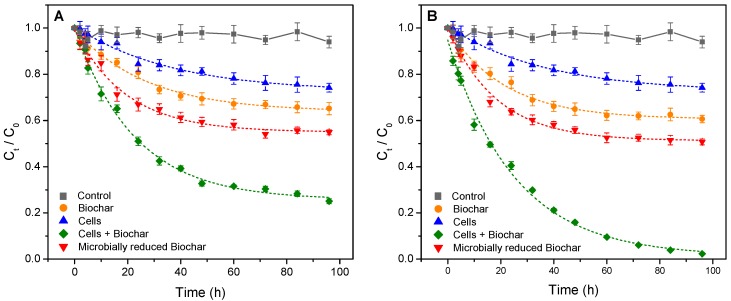
OG (Orange G) removal curves in different treatment systems. Biochars in (**A**,**B**) were prepared using peanut stalks from the plot without Fe addition and from the plot with Fe addition, respectively. The dashed lines show nonlinear least-squares regression fits to a first-order rate law.

The concentration of Orange G in the solution in the control treatment did not show significant fluctuation with the incubation time (Figure 2). This result indicated that no spontaneous degradation removal of Orange G occurred during incubation. In the presence of BCNF or BCWF, the concentration of Orange G in the solution decreased gradually with increasing incubation time (Figure 2). This result indicated that Orange G can be removed partly by using biochar alone.

**Figure 3:**
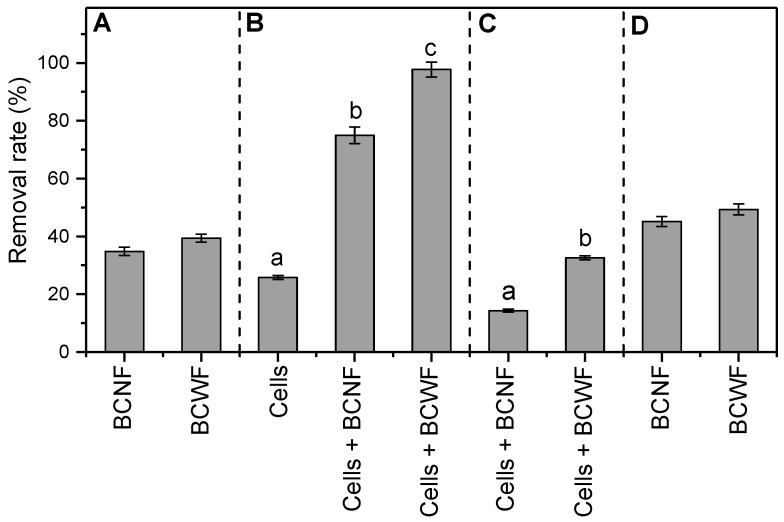
OG removal rates in different treatment systems. (**A**), Removal rates of Orange G in the presence of biochars alone. (**B**), Removal rates of Orange G in the presence of cells alone and in the presence of cells and biochars. (**C**), Rates of the microbially reductive removals of Orange G mediated by biochars. (**D**), Removal rates of Orange G in the presence of microbially reduced biochars. BCNF and BCWF indicate the biochars prepared using peanut stalks from the plot without Fe addition and from the plot with Fe addition, respectively. In (**B**,**C**), means followed by the different lowercase letters indicate significantly different at a level of *P* < 0.05.

Aniline as a possible reduction product of Orange G was not detected in the suspension in the presence of BCNF or BCWF. This result indicated that BCNF and BCWF possibly functioned as an adsorbent in part removing the Orange G in the solution in the presence of biochar alone. The specific surface area, pore structure, particle size, surface functional groups, and pH of the biochars are the main physicochemical parameters that govern their abilities to adsorb organic pollutants [19]. BCNF and BCWF possessed the same properties in these parameters (Table 2), which may be responsible for the result that these two biochars did not exert significant difference in the adsorbing removal rate of Orange G in the solution (Figure 3A).

### 3.3. Stimulated Microbial Reduction of Orange G by Biochars as an Extracellular Electron Shuttle

**Figure 4:**
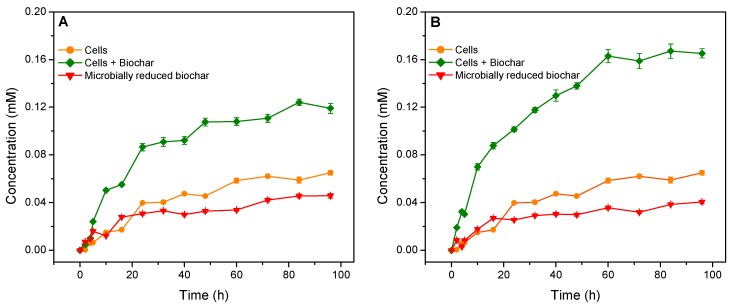
Concentration changes of aniline detected in different treatment systems with incubation time. Biochars in (**A**,**B**) were prepared using peanut stalks from the plot without Fe addition and from the plot with Fe addition, respectively.

In the presence of *S. oneidensis* MR-1, a slightly decreasing trend in the concentration of Orange G in the solution and a gently increasing trend in aniline concentration in the suspension with increasing incubation time were observed (Figure 2 and Figure 4). These results indicated that *S. oneidensis* MR-1 is a kind of Fe-reducing bacteria that has the potential to reduce Orange G to aniline, which is consistent with the previous viewpoint [29,30]. In the presence of *S. oneidensis* MR-1 and biochar, the removal rate of Orange G in the solution significantly accelerated (Figure 3B). The rate of the microbially reductive removal of Orange G mediated by biochar, which was calculated by subtracting the rate of adsorbing removal of Orange G by the biochars and the rate of the direct microbially reductive removal of Orange G in the presence of *S. oneidensis* MR-1 alone from the rate of Orange G removal in the presence of *S. oneidensis* MR-1 and biochar, was remarkably greater than the rate of Orange G removal by using *S. oneidensis* MR-1 alone (Figure 3C). Moreover, the concentrations of aniline in the suspensions in the presence of *S. oneidensis* MR-1 and biochars were notably higher than that in the presence of *S. oneidensis* MR-1 alone (Figure 4). These results indicated that BCNF and BCWF possessed the potential to stimulate the microbial reduction of Orange G to aniline in the solution by acting as an extracellular electron shuttle.

The rate of microbially reductive removal of Orange G mediated by BCNF was significantly lower than that mediated by BCWF (Figure 3C). Moreover, the concentration of aniline in the suspension in the presence of *S. oneidensis* MR-1 and BCNF was remarkably lower than that in the presence of *S. oneidensis* MR-1 and BCWF (Figure 4). These results suggested that BCNF and BCWF may employ different extracellular electron shuttling mechanisms to stimulate the microbial reduction of Orange G to aniline.

### 3.4. Role of Biochar as the Battery and Conductor in Stimulating the Microbial Reduction of Orange G

**Figure 5:**
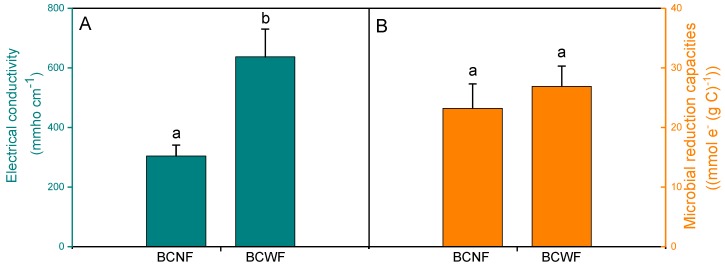
Comparisons of electron conductivity (**A**) and microbial reduction capacity (**B**) between biochars prepared using peanut stalks from the plot without Fe addition (BCNF) and from the plot with Fe addition (BCWF). Mean values with the different lowercase letters represent significant difference between BCNF and BCWF at *P* < 0.05.

Biochar has been promoted as a conductor and multicycle battery to mediate the electron transfer from cells to terminal electron acceptors [20,28,31]. The role of the battery mechanism of biochar in mediating electron shuttle depends on the charging and discharging capacities of its surface redox-active functional groups [22], which were indirectly estimated by the microbial reduction capacity of the biochar in this study. No significant difference was observed in microbial reduction capacities between BCNF and BCWF (Figure 5), possibly due to the identical distributions of the redox-active functional groups (e.g., quinones) in the surfaces of BCNF and BCWF. This result indicated that a high Fe content of the precursor did not promote the charging and discharging capacities of the produced biochar.

The conductor performance of biochar, which refers to the ability of the biochar to directly transfer electrons [22], was indirectly evaluated by its EC in this study. Results revealed that the EC of BCWF was notably greater than that of BCNF (Figure 5). This finding indicated that a high Fe content of the precursor can significantly enhance the conductor performance of the produced biochar. Analogous to the electron-carrying heme in the outer membrane cytochromes of microorganisms [32,33], the abundance of Fe in the BCWF formed an electron transfer conduit that was favorable for directly transferring electrons. In addition, the presence of Fe species in the PSWF catalyzed the growth of C nanofibers on the surface of the BCWF during pyrolysis [34,35], thereby increasing the conductor performance of the BCWF.

**Figure 6:**
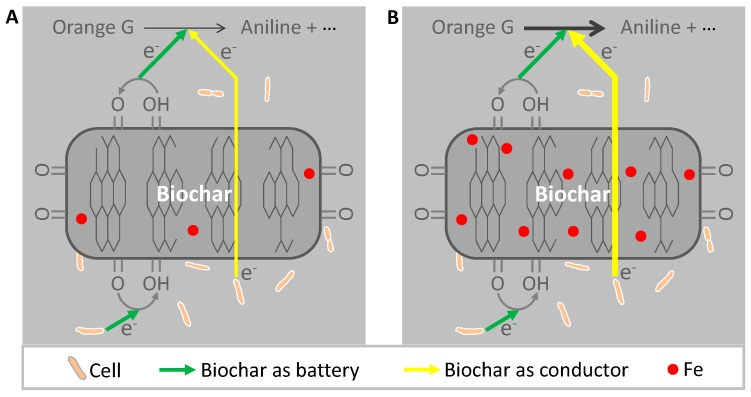
Schematic diagram of the biochar internal pathways for electron transfer in microbial reduction of Orange G to aniline mediated by biochars prepared using peanut stalks from the plot without Fe addition (**A**) and from the plot with Fe addition (**B**). Green arrows represent the charging and discharging cycles of the biochar battery mechanism, and yellow arrows represent the biochar conductor mechanism in which electrons transfer directly through conductive domains.

Therefore, the conductor mechanism, rather than the battery mechanism, may be responsible for the more stimulated microbial reduction of Orange G to aniline by BCWF rather than by BCNF (Figure 6). To further verify this speculation, the treatments added with the BCNF and BCWF reduced by *S. oneidensis* MR-1 were conducted. Results revealed that both the removal rate of Orange G in the solution and the concentration of aniline detected in the suspension in the presence of the microbially reduced BCNF were not significantly different from those in the presence of the microbially reduced BCWF (Figure 3D and Figure 4). This result not only indicated that biochars as battery possessed the potential to stimulate the microbial reduction of Orange G to aniline but also further confirmed that the phenomenon of BCWF being more advantageous than BCNF in stimulating microbial reduction of Orange G to aniline was mainly attributed to the difference in conductor performances rather than in battery performances between these two biochars.

### 3.5. Implications for the Future

Given the abundant functionality of biochars, much attention has been dedicated to the applications of biochars in the field of environmental protection, such as organic and inorganic pollutant removal [36,37,38,39]. Given the positive association of stimulation effectiveness of biochars in the microbial reduction of azo dyes with their conductor and battery performances, it is expected that the produced biochars are rapid in directly transferring electrons and in the charging and discharging cycles of their surface redox-active functional groups. It has been demonstrated that the lower H/C and O/C ratios of the biochar enhanced its conductor performance [22], by which electrons can be directly transferred rapidly from the cells to the terminal electron acceptors. Although the features of the element composition in biochars can be fine-tuned by adapting the pyrolysis conditions (e.g., heating rate and temperature) [40,41], exogenetic elements may only be doped on the surface of the biochar and element-doping requires a series of specialized processes, which may lead to the fact that the element-rich biochar cannot exert the greatest conductor mechanism and that it is expensive. The result of this study revealed a positive link between the conductor performance of biochar and Fe content of the precursor. Thus, further efforts need to be focused on the search for a natural biomass with a high Fe content to allow the produced biochar to have a wide application in stimulating the microbial reduction of redox-active pollutants.

Biochar not only provides a way to turn waste into treasure for agricultural and forestry waste such as straw, but also provides an effective means for eco-environmental management. The industrialization of biochar technology is of great significance for controlling non-point source pollution, promoting the sustainable development of the ecological environment and ensuring national food and energy security [42]. Therefore, the development of biochar industrialization is the current inevitable trend. The superior performance of biochar in environmental restoration is a prerequisite for promoting its industrialization. Our results provide evidence for the first time that a high Fe content of biomass can significantly enhance the conductor performance of the produced biochar, which is of significant importance in environmentally relevant redox reactions of pollutants, including azo dyes selected as the target in this study and other redox-active pollutants [43]. This indicates that the use of iron-rich biomass to prepare biochar is an alternative, low-cost, and eco-friendly method that can promote the industrialization of biochar, because it can weaken the modification process after biochar preparation and thus correspondingly save part of the cost for preparing biochar. Iron ore resources are very rich in China, and the resulting land pollution is also creating a serious situation [44]. We recommend planting plants with a high resistance to heavy metals in iron ore areas, not only to restore the polluted land through phytoremediation, but also to provide iron-rich biomass for the preparation of biochar, which will be beneficial to developing the biochar industry with Chinese characteristics and in line with China’s national conditions. In addition, the cost-benefit analysis regarding applications of the biochar produced with Fe-rich-biomass pyrolysis should be considered in future studies for developing the biochar industry.

## 4. Conclusions

The results of this study demonstrated that Orange G can be effectively reduced by *S. oneidensis* MR-1 in the presence of biochars. Both the conductive domains and charging and discharging of the surface functional groups in the biochars played crucial roles in the microbial reduction of Orange G to aniline. The better performance of BCWF over BCNF in stimulating microbial reduction of Orange G was mainly attributed to the higher conductor performances in BCWF than in BCNF. Our results revealed for the first time that the biochar produced by pyrolysis of Fe-rich biomass can promote the extracellular electron shuttle by improving conductor performance. The findings of this study can provide aid in utilizing biochar from iron-rich-biomass pyrolysis to alleviate the environmental pollution caused by azo dyes.

## Figures and Tables

**Table 1:** Element contents of the peanut stalks obtained from the plot without Fe addition and the plot with Fe addition. Mean (± SE, n = 5).

Straw Type	Fe (mg kg^−1^)	C (g kg^−1^)	N (g kg^−1^)	H (g kg^−1^)	O (g kg^−1^)
PSNF	18.2 ± 1.7 a	482 ± 38	32.4 ± 3.0	43.2 ± 4.2	329 ± 29
PSWF	79.7 ± 6.0 b	464 ± 41	35.2 ± 2.8	40.5 ± 3.4	310 ± 25

Notes: PSNF and PSWF indicate the peanut stalks obtained from the plot without Fe addition and the plot with Fe addition, respectively. Mean values with the different lowercase letters represent significant difference between PSNF and PSWF at *P* < 0.05.

**Table 2:** Physicochemical properties of BCNF and BCWF. Mean (± SE, n = 5).

Parameter	BCNF	BCWF
C (%)	53.2 ± 3.7	56.7 ± 4.0
H (%)	3.67 ± 0.24	3.52 ± 0.29
N (%)	1.87 ± 0.16	1.98 ± 0.15
O (%)	23.4 ± 2.8	26.1 ± 2.1
P (%)	0.38 ± 0.05	0.31 ± 0.06
K (%)	1.12 ± 0.17	0.97 ± 0.11
Ca (%)	8.53 ± 0.61	8.11 ± 0.57
Mg (%)	6.12 ± 0.48	5.66 ± 0.52
Fe (mg kg^−1^)	548 ± 43a	3578 ± 210b
Co (mg kg^−1^)	1.17 ± 0.08	1.32 ± 0.18
Cu (mg kg^−1^)	27.3 ± 2.3	23.4 ± 3.5
Zn (mg kg^−1^)	17.5 ± 1.2	15.7 ± 1.5
Mo (mg kg^−1^)	0.34 ± 0.06	0.41 ± 0.04
Ni (mg kg^−1^)	3.11 ± 0.41	3.43 ± 0.34
pH	6.84 ± 0.11	7.21 ± 0.27
Ash (%)	19.3 ± 1.4	21.7 ± 2.0
Specific surface area (m^2^ g^−1^)	279 ± 23	305 ± 34

Notes: BCNF and BCWF indicate the biochars prepared using peanut stalks from the plot without Fe addition and from the plot with Fe addition, respectively. Mean values with the different lowercase letters represent significant difference between BCNF and BCWF at *P* < 0.05.
